# Supplementary figures and images for: Ciliary Neurotrophic Factor Modulates Multiple Downstream Signaling Pathways in Prostate Cancer Inhibiting Cell Invasiveness
Source: Cancers (Basel). 2022 Nov 30;14(23):5917. doi: 10.3390/cancers14235917 (PMC9739171; doi:10.3390/cancers14235917)

Figure 3a

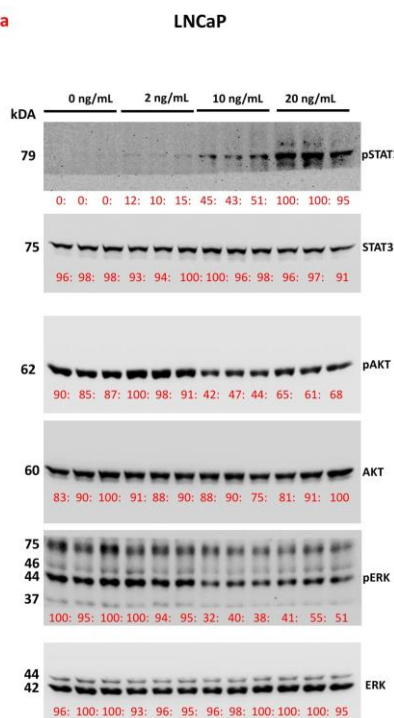

Figure 3b

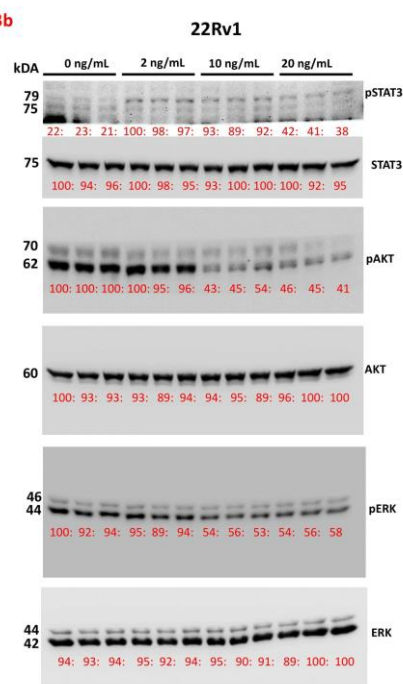

Figure 4a

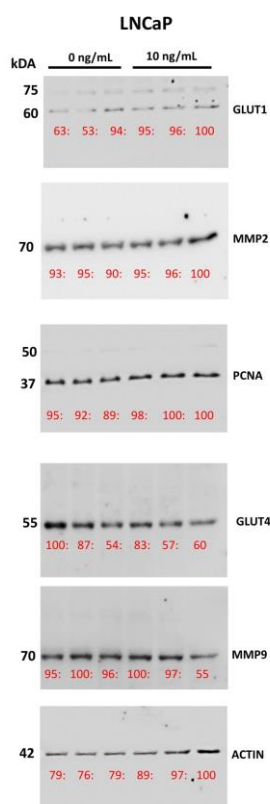

Figure 4b

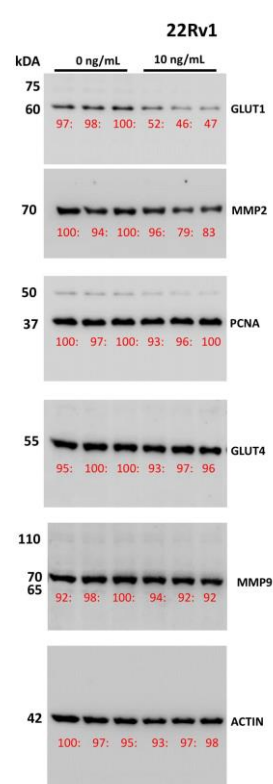

Figure S1. Original images of immunoblotting data.

Supplement: Supplementary file 1 [file cancers-14-05917-s001.zip › cancers-1998365-supplementary.pdf]
